# Development of visible light‐sensitive human neuropsin (OPN5) via single amino acid substitution

**DOI:** 10.1002/1873-3468.70130

**Published:** 2025-08-13

**Authors:** Yusuke Sakai, Richard J. McDowell, Robert J. Lucas

**Affiliations:** ^1^ Division of Neuroscience and Centre for Biological Timing School of Biological Sciences, Faculty of Biology Medicine and Health, University of Manchester UK

**Keywords:** GPCR, neuropsin, opsin, optogenetics, spectral tuning

## Abstract

Neuropsin (Opn5), a UV‐sensitive ‘non‐visual’ opsin, has the potential to be used as optogenetic tools applicable to tissues outside of the eye because of its broad expression. However, its sensitivity to poorly tissue‐penetrating UV light poses challenges for its application. In this study, we focused on human OPN5 (hOPN5) to identify amino acid(s) responsible for the UV sensitivity. Sequence alignment across UV‐sensitive Opn5s identified a conserved lysine residue (Lys91) at a position implicated in spectral tuning in invertebrate opsins. Substitution of this residue with neutral or acidic amino acids caused substantial shifts in spectral sensitivity towards visible wavelengths. Our findings identify Lys91 as a key spectral tuning site in hOPN5 and provide visible‐light‐sensitive versions as a candidate for optogenetic applications.

**Impact statement**

A “non‐visual” opsin, Opn5, is the only UV‐sensitive opsin in human. In this study, we identified, for the first time, a key tuning site responsible for the UV sensitivity of hOPN5. In addition to its impact on the opsin molecular studies, this finding could pave the way for the development of novel visual‐sensitive Opn5‐based optogenetic tools.

## Graphical abstract

The present study determines a key amino acid residue, Lys91, for defining UV sensitivity of human OPN5. Heterologous action spectroscopy of the wild type and K91 mutants of OPN5 in HEK293T cells reveals that substitution of Lys91 with neutral (alanine) or acidic amino acids (glutamic or aspartic acids) causes substantial shifts in spectral sensitivity (55–63 nm) toward visible wavelengths.

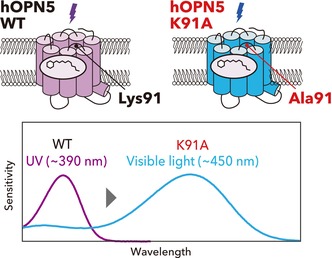

## Abbreviations

**GPCRs**, G protein‐coupled receptors

**hOPN5**, human opsin‐5, neuropsin

**HEK293T**, Human embryonic kidney 293T

Animal opsins are light‐sensitive G protein‐coupled receptors (GPCRs) that convert light into intracellular G protein signalling. In vertebrates, opsins extend beyond the well‐studied rod and cone photopigments underlying vision to encompass various ‘non‐visual’ opsins identified from tissues outside photoreceptor cells. Neuropsin (opsin‐5, hereafter referred to as Opn5) is a type of non‐visual opsin first identified in the genomes of humans and mice [1]. Mouse Opn5 is expressed in a wide range of tissues including the brain and skin [1, 2] and has been reported to mediate various light‐dependent physiologies such as direct photoentrainment of local circadian clocks in the cornea [3] and skin [4] and the violet‐light‐dependent suppression of brown adipose tissue (BAT) activity [5]. In non‐mammalian vertebrates, quail Opn5 is expressed in the hypothalamus paraventricular organ (PVO) where it contributes to the regulation of the seasonal cycle of reproduction [6]. In the Japanese rice fish (medaka), Opn5 expressed in pituitary melanotrophs regulates the short‐wavelength‐light‐dependent release of melanocyte‐stimulating hormone (MSH) and subsequent pigmentation in the skin [7].

In addition to such physiological importance, the Opn5 family has also attracted attention for its potential for generating optogenetic tools that regulate intracellular G protein signalling with light. A recent study has shown that human OPN5 (hOPN5) selectively activates the Gq‐type G protein without promiscuous activation of other G proteins such as Gi [8] which most other Gq‐coupled opsins, including melanopsins [9, 10] and jumping spider Rh1 [11, 12] activate. hOPN5, as a Gq‐selective optogenetic tool, has been applied *in vivo* to murine cardiomyocytes to modulate heart beat rate and smooth muscle cells in various organs, such as the small intestine and stomach in mice, to induce muscle contraction with light via Gq signalling pathways [8, 13].

Although hOPN5 is becoming increasingly valuable in the field of optogenetics, several challenges remain to be addressed for its widespread application. One of the most important problems is that it is primarily sensitive to UV light, which has much lower penetration of biological tissues than visible light due to higher absorbance and scattering. Homologues of hOPN5 from across the vertebrates (the so called ‘Opn5m’ sub‐family; Fig. S1) form UV‐absorbing pigments with λ_max_ at 360–380 nm upon binding 11‐*cis* retinal in the dark [2, 14]. The highly conserved UV sensitivity among these Opn5m opsins suggests the existence of shared amino acid residue(s) responsible for the UV sensitivity. In this study, we set out to identify such responsible amino acid residue(s) for the UV sensitivity of hOPN5. We applied sequence alignment and homology modelling to identify conserved residues close to the chromophore binding pocket with potential to provide UV sensitivity. We then applied site‐directed mutagenesis and heterologous action spectroscopy [9, 15] in HEK293 cells to determine their involvement in this property. We find that a lysine residue, Lys91 (bovine rhodopsin amino acid numbering), in helix II is necessary to make hOPN5 UV‐sensitive. Based on this result, we discuss the potential use of hOPN5 K91 mutants as visible light‐sensitive Gq‐manipulating optogenetic tools.

## Material and methods

### Construction of opsin expression vectors

mRNA sequence of hOPN5 was obtained from NCBI GenBank (NM181744). We designed sequences containing the open reading frames of wild type and mutants of hOPN5 tagged with rho 1D4 epitope sequences (ETSQVAPA) at the C‐terminus. These DNA sequences were then synthesised using the gene synthesis service of Twist Bioscience. The synthesised DNA fragments were inserted into the HindIII/NotI‐digested pcDNA3.1 mammalian expression vector (Thermo Fisher Scientific, Waltham, MA, USA) using NEB HiFi Assembly (New England Biolabs, Ipswich, MA, USA).

### Cell line and treatment

Human embryonic kidney 293T cells (RRID: CVCL_0063) were purchased from ATCC (CRL‐11268). The cells have been authenticated and confirmed to be free of mycoplasma contamination by the supplier. HEK293T cells were cultured in Dulbecco's modified Eagles medium—high glucose (Merck, Darmstadt, Germany) with 10% foetal bovine serum (FBS) and 1% Penicillin/Streptomycin under mycoplasma‐free conditions and were used for experiments at a low passage number.

### Bioluminescence‐based Ca^2+^ measurements in HEK293T cells

HEK293T cells were seeded on 12‐well plates (~500 000 cells per well) and transiently transfected ~24 h later with expression vectors for each target opsin (500 ng) and genetically encoded bioluminescent Ca^2+^ reporter, mtAequorin (500 ng) using Lipofectamine 2000 Transfection Reagent (Thermo Fisher Scientific) as described previously [9, 15]. Four to six hours after transfection, 10 μm 11‐*cis* retinal (National Eye Institute, National Institutes of Health, Bethesda, MD, USA) was added to the cells. The cells were subsequently added to white clear‐bottom 96‐well plates for incubation at 37°C overnight. The following day, the cells were incubated in Leibovitz's L‐15 Medium (Thermo Fisher Scientific) containing 10 μm Coelenterazine‐h (Promega, Madison, WI, USA), 10 μm 11‐*cis* retinal, and 1% FBS in the dark at room temperature for 2 h before recording luminescence. For each recording, baseline luminescence was recorded for 10 s and then the cells were stimulated with a 1 s light flash of varying intensities (12.5 to 16 log_10_photons·cm^−2^·s^−1^) at one of distinct wavelengths (365, 385, 405, 435, 460, 490, and 525 nm). Luminescence signals were measured every 2 s using a plate reader (Optima FLUOStar, BMG Labtech, Ortenberg, Germany) and an external light source (CoolLED PE‐4000, CoolLED, Andover, UK) via a liquid light‐guide with a set of neutral density filters (Thorlabs, Newton, NJ, USA) used to illuminate the cells.

### Calculation of wavelength of maximum sensitivity for each opsin

Based on irradiance‐response curves obtained from the aequorin Ca^2+^ assay, we determined the λ_max_ values for each opsin using a nonlinear curve fitting with a bootstrapping approach as described previously [15]. Briefly, we first calibrated the measured irradiance (log_10_photons·cm^−2^·s^−1^) according to the Govardovskii opsin pigment template [16] with a given λ_max_ value (namely, each measured irradiance‐response curve was multiplied by a template absorption spectrum having a given λ_max_ value) and obtained the effective light intensity. Then, a 5‐parameter logistic model was fitted with the normalised Ca^2+^ increase as the dependent variable and the effective light intensity as the independent variable using the *drm* function from the *drc* package in R. The residual sum of squares (RSS) extracted from each fitting was passed to the optimisation algorithm (*optim* function in R using the Brent search method) to identify the λ_max_ value (within a 350–550 nm range) that would minimise the RSS. Bootstrapping (100 iterations) was applied, in which the optimisation procedure was performed for each dataset constructed based on resampling with replacement from the original dataset, and the average value of the 100 replicates was used as the λ_max_ estimate. For λ_max_ estimation, we used irradiance‐response datasets at 365, 385, 405, and 435 nm for wild‐type hOPN5 and 385, 405, 435, 460, and 490 nm for K91 mutants hOPN5, where Ca^2+^ responses were nearly saturated. All analyses were performed in R version 4.4.3 [17].

### Molecular phylogenetic tree inference

Amino acid sequences of opsins obtained from NCBI GenBank database were aligned using MAFFT [18] and trimmed by TrimAl [19] with ‘gappyout’ function. The maximum likelihood tree was reconstructed using RAxML‐NG v.1.2.0 [20] assuming the LG + I + G4 + FC model, which was selected using ModelTest‐NG v.0.2.0 [21]. The ML branch supports were obtained by 500 bootstrap resamplings.

## Results

**Fig. 1:**
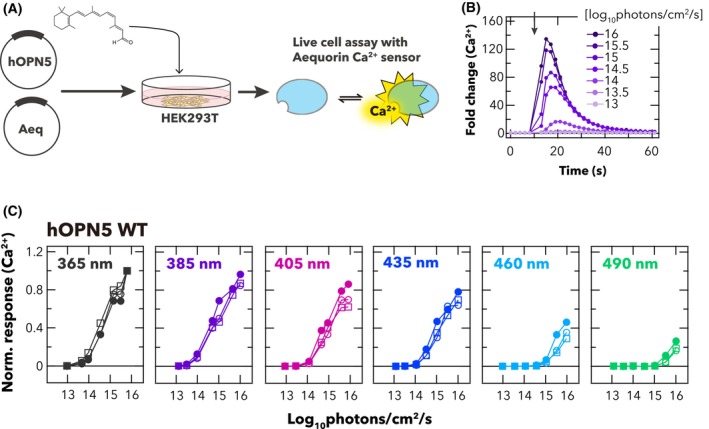
Measurement of light‐induced Ca^2+^ response in HEK293T cells transfected with wild‐type hOPN5. (A) Schematic of bioluminescence‐based Ca^2+^ assay using aequorin Ca^2+^ biosensor. HEK293T cells were transfected with hOPN5‐ and aequorin‐expression vectors and incubated with 11‐*cis* retinal. Bioluminescence from aequorin Ca^2+^ sensor was recorded using a plate reader. (B) Time course of light‐evoked Ca^2+^ increase in the hOPN5‐expressing cells. Luminescence values were normalised to the baseline (=1). The cells were stimulated with various intensities of 385‐nm light (1 s duration) at the time indicated by the black arrow in the graph. (C) Irradiance‐response curves of wild‐type hOPN5‐expressing cells for six spectrally distinct light stimuli (numerical values at top left depict wavelength at peak photon flux). The peak Ca^2+^ responses achieved for each stimulus were normalised to the maximum value observed across all wavelength & intensity combinations (as 1) for each independent experiment. Each replicate is indicated by different shapes of symbols (*n* = 3).

We first measured light‐dependent Ca^2+^ increases in wild‐type hOPN5‐transfected HEK293T cells via an aequorin‐based bioluminescent assay (Fig. 1A) and confirmed that it induced light intensity‐dependent Ca^2+^ increases in the cells (Fig. 1B). The irradiance‐response curves for six spectrally distinct stimuli showed that the wild‐type hOPN5 had a higher sensitivity to stimuli rich in UV than visible light (Fig. 1C). This finding confirms that hOPN5 forms UV‐sensitive pigments in the HEK293T cell environment to drive Gq‐mediated Ca^2+^ responses upon light absorption, as previously reported [2, 8, 14].

**Fig. 2:**
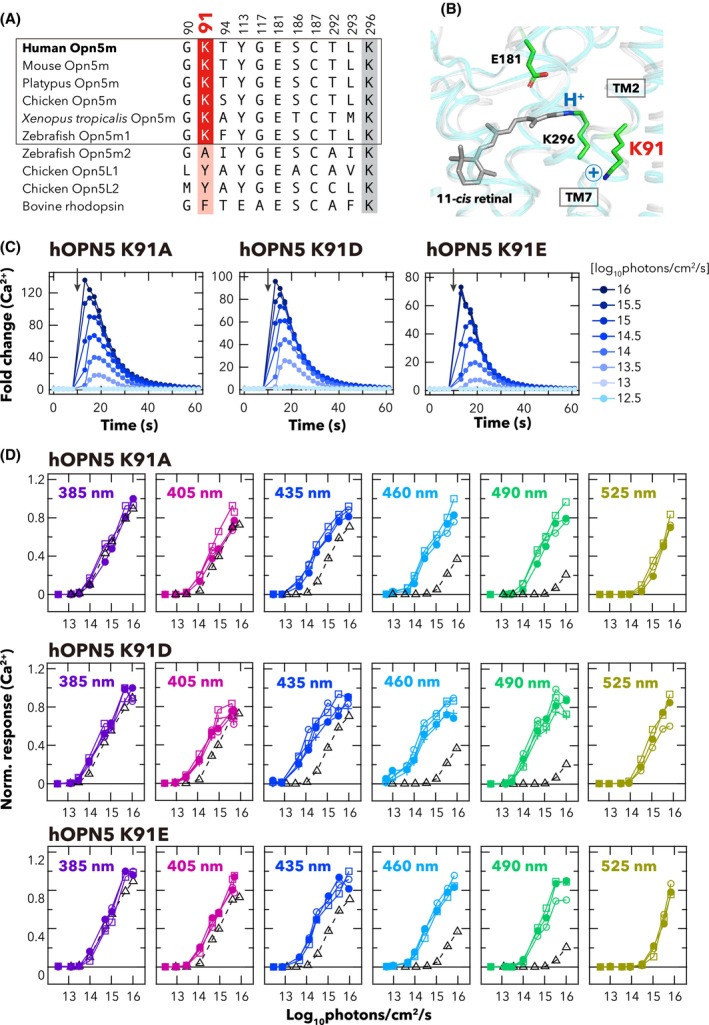
Lys91 mutants hOPN5 show enhanced sensitivity to visible light. (A) Comparison of amino acid residues near the retinylidene Schiff base among opsins in Opn5 group and bovine rhodopsin. The GenBank accession numbers for each opsin are provided in Fig. S1. (B) The Schiff base environment in hOPN5 depicted according to Alpha‐fold3 3D model of hOPN5 (cyan) and X‐ray crystal structure of bovine rhodopsin (PDBID: 1U19, grey). (C) Time courses of light‐evoked Ca^2+^ responses in hOPN5 K91 mutants‐expressing cells. Luminescence values were normalised to the baseline (=1). The cells were stimulated with various intensities of 435‐nm light (1 s duration) at the time indicated by the black arrow in the graph. (D) Irradiance‐response curves of hOPN5 K91A, K91D and K91E are shown for six spectrally distinct light stimuli (numerical values at top left depict wavelength at peak photon flux). The peak Ca^2+^ responses achieved for each stimulus were normalised to the maximum value observed across all wavelength & intensity combinations (as 1) for each independent experiment. Each replicate is indicated by different shapes of symbols (*n* = 3–4). Black open triangles with dashed lines indicate the mean values of wild‐type hOPN5 (reproduced from Fig. 1C).

To determine the amino acid residue(s) responsible for the UV sensitivity of hOPN5, we searched amino acids which are conserved across the Opn5m sub‐family and located near the Schiff base based upon AlphaFold3 structural modelling [22]. The most promising candidate was a conserved Lys (position 91) in helix II of Opn5s (Fig. 2A,B). Replacement of Lys residues at an equivalent location in UV‐sensitive *Drosophila melanogaster* Rh3 and Rh7 opsins and *Platynereis dumerilii* c‐Opsin1 has successfully produced shifts to visible wavelength sensitivity [23, 24, 25].

**Fig. 3:**
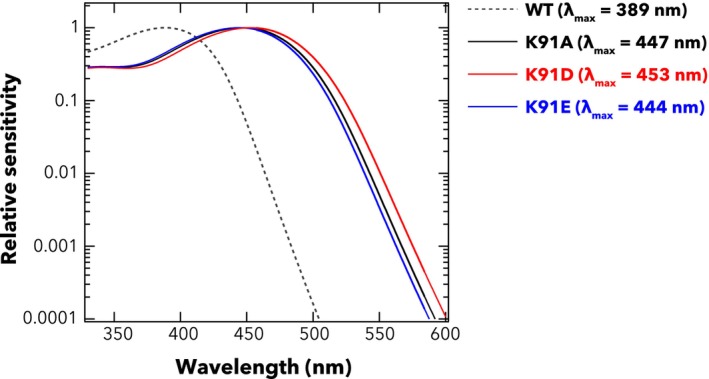
Predicted spectral sensitivity functions for wild type and Lys91 mutants of hOPN5. Curves show Govardoskii opsin pigment templates that best predict the set of irradiance‐response curves depicted in Figs 1 and 2 for WT (black dotted), K91A (black), K91D (red) and K91E (blue) mutants.

To determine the contribution of Lys91 to UV sensitivity of hOPN5, we substituted the residue with amino acids having neutral (alanine) or negatively charged (glutamic/aspartic acids) side chains and examined the spectral sensitivity of the resultant K91 mutants. We confirmed that the cells expressing K91 mutants (K91A, K91D, and K91E) exhibited an increase in intracellular Ca^2+^ following 435‐nm light irradiation, with peak response amplitudes ranging from 80‐ to 130‐fold above baseline levels (Fig. 2C), showing the mutants remained functionally sufficient. Construction of irradiance‐response curves for spectrally distinct stimuli showed that the K91 mutants had higher sensitivity to visible light compared to the wild type (Fig. 2D). The λ_max_ values of wild type and K91A mutants of hOPN5 were estimated by applying a nonlinear optimisation algorithm with bootstrapping to the experimentally obtained light intensity‐Ca^2+^ response dataset [15] (see Fig. S2 and Material and Methods section for details). The estimated λ_max_ values were 389 nm for wild type (similar to that reported elsewhere), while K91A, K91D, and K91E mutants of hOPN5 had λ_max_ around 447, 452, and 444 nm respectively (Fig. 3). These results indicate that a single amino acid mutation at Lys91 is sufficient to make hOPN5 visible light sensitive.

## Discussion

The visible light shift we observed in the K91A, K91D & K91E mutants implicates Lys91 as a critical residue in defining UV sensitivity for human OPN5. Recent report of a similar effect for Lys91 mutants of *Xenopus* Opn5m indicates that this is a general feature of this opsin sub‐family [26]. Opsins can absorb visible light only when the chromophore retinal is covalently bound to the opsin via a protonated Schiff base. Consequently, the most parsimonious explanation for the role of Lys91 in UV sensitivity is that the positively charged side chain of this residue destabilises the Schiff base protonation. Such a mechanism has been suggested to explain the contribution of Lys residues to UV sensitivity at sites 90 and 94 in *D. melanogaster* Rh3 and Rh7, respectively, and *P. dumerilii* c‐Opsin1 [23, 24, 25]. Moreover, in teleost parapinopsins (non‐visual opsins phylogenetically close to vertebrate rod and cone opsins), introduction of helix II from UV‐sensitive PP1 is sufficient to provide a λ_max_ in the UV light region to the visible light (blue)‐sensitive PP2 [27] and also, a report has demonstrated that G90K mutation in bovine rhodopsin shifts the λ_max_ to the UV region (384 nm) [28]. Thus, across diverse opsins, residues in helix II appear to define UV sensitivity. However, according to the Alpha‐fold3 3D model of hOPN5 aligned with the crystal structure of bovine rhodopsin (Fig. 2B), the side chain of Lys91 in hOPN5 is about 8 Å away from the Schiff base. It remains to be determined how such a distant residue could destabilise Schiff base protonation. Furthermore, sequence alignments across more distantly related Opn5s (Fig. S1) reveal UV‐sensitive opsins (Opn5m2 and Opn5L2) [29, 30] that lack Lys91 (Fig. 1A), indicating that these Opn5 homologues have different spectral tuning mechanisms from Opn5m for their UV sensitivity.

The possible immune response against optogenetic protein itself has been reported as well as against AAV‐mediated gene transfer [31]. Mammalian non‐visual opsins including Opn3, Opn4, and Opn5 [32] that exhibit broad tissue expression represent an attractive strategy to reduce the likelihood of such immune responses; therefore, they have a significant advantage for both experimental and therapeutic optogenetic applications. hOPN5 has generated particular interest due to its specific activation of Gq‐type G proteins [8, 33] and successful *in vivo* applications of hOPN5 have been demonstrated, such as light‐dependent control of muscle contraction across a wide range of muscle tissues [8, 13]. The visual light‐sensitive versions of hOPN5 in this study have the potential to be applied to deep tissues where UV light penetration is limited and thus could be a good template upon which to develop general Gq‐manipulating optogenetic tools. As there has been accumulating knowledge about spectral sensitivity tuning within the visible light range [34, 35, 36], engineering of further red‐shifted mutants of hOPN5 could prove fruitful in future studies.

## Author contributions

YS: conceptualisation, investigation, formal analysis, data curation, visualisation, writing— original draft, writing—review and editing. RJM: methodology, investigation, writing—review and editing. RJL: conceptualisation, writing—original draft, writing—review and editing, supervision, project administration, funding acquisition.

## Peer review

The peer review history for this article is available at https://www.webofscience.com/api/gateway/wos/peer‐review/10.1002/1873‐3468.70130.

## Supporting information


**Fig. S1.** Maximum likelihood tree of Opn5 group.
**Fig. S2.** Estimation of spectral sensitivities for wild type and Lys91 mutants of hOPN5 by a nonlinear optimisation with bootstrap samplings.

## Data Availability

All the primary data that support the results of this study are available from the corresponding authors upon reasonable request.

## References

[1] Tarttelin EE , Bellingham J , Hankins MW , Foster RG and Lucas RJ (2003) Neuropsin (Opn5): a novel opsin identified in mammalian neural tissue. FEBS Lett 554, 410–416. 14623103 10.1016/s0014-5793(03)01212-2

[2] Kojima D , Mori S , Torii M , Wada A , Morishita R and Fukada Y (2011) UV‐sensitive photoreceptor protein OPN5 in humans and mice. PLoS One 6, e26388. 22043319 10.1371/journal.pone.0026388 PMC3197025

[3] Buhr ED , Yue WWS , Ren X , Jiang Z , Liao H‐WR , Mei X , Vemaraju S , Nguyen M‐T , Reed RR , Lang RA *et al*. (2015) Neuropsin (OPN5)‐mediated photoentrainment of local circadian oscillators in mammalian retina and cornea. Proc Natl Acad Sci U S A 112, 13093–13098. 26392540 10.1073/pnas.1516259112 PMC4620855

[4] Buhr ED , Vemaraju S , Diaz N , Lang RA and Van Gelder RN (2019) Neuropsin (OPN5) mediates local light‐dependent induction of circadian clock genes and circadian photoentrainment in exposed murine skin. Curr Biol 29, 3478–3487. 31607531 10.1016/j.cub.2019.08.063 PMC6814305

[5] Zhang KX , D'Souza S , Upton BA , Kernodle S , Vemaraju S , Nayak G , Gaitonde KD , Holt AL , Linne CD , Smith AN *et al*. (2020) Violet‐light suppression of thermogenesis by opsin 5 hypothalamic neurons. Nature 585, 420–425. 32879486 10.1038/s41586-020-2683-0 PMC8130195

[6] Nakane Y , Ikegami K , Ono H , Yamamoto N , Yoshida S , Hirunagi K , Ebihara S , Kubo Y and Yoshimura T (2010) A mammalian neural tissue opsin (opsin 5) is a deep brain photoreceptor in birds. Proc Natl Acad Sci USA 107, 15264–15268. 20679218 10.1073/pnas.1006393107 PMC2930557

[7] Fukuda A , Sato K , Fujimori C , Yamashita T , Takeuchi A , Ohuchi H , Umatani C and Kanda S (2025) Direct photoreception by pituitary endocrine cells regulates hormone release and pigmentation. Science 387, 43–48. 39745961 10.1126/science.adj9687

[8] Wagdi A , Malan D , Sathyanarayanan U , Beauchamp JS , Vogt M , Zipf D , Beiert T , Mansuroglu B , Dusend V , Meininghaus M *et al*. (2022) Selective optogenetic control of Gq signaling using human Neuropsin. Nat Commun 13, 1765. 35365606 10.1038/s41467-022-29265-w PMC8975936

[9] Bailes HJ and Lucas RJ (2013) Human melanopsin forms a pigment maximally sensitive to blue light (λ_max_ ≈ 479 nm) supporting activation of G(q/11) and G(i/o) signalling cascades. Proc Biol Sci 280, 20122987. 23554393 10.1098/rspb.2012.2987 PMC3619500

[10] McDowell RJ , Rodgers J , Milosavljevic N and Lucas RJ (2022) Divergent G‐protein selectivity across melanopsins from mice and humans. J Cell Sci 135, jcs258474. 35274137 10.1242/jcs.258474 PMC8977054

[11] Varma N , Mutt E , Mühle J , Panneels V , Terakita A , Deupi X , Nogly P , Schertler GFX and Lesca E (2019) Crystal structure of jumping spider rhodopsin‐1 as a light sensitive GPCR. Proc Natl Acad Sci U S A 116, 14547–14556. 31249143 10.1073/pnas.1902192116 PMC6642406

[12] Iwasaki M , Sakai Y , Shirata T , Sugihara T , Koyanagi M and Terakita A (2025) Characterization of anthozoan‐specific opsins from a reef‐building coral, *Acropora tenuis*, as Gq‐coupled opsins. Zoolog Sci 42, 196–205. 40184198 10.2108/zs240085

[13] Zipf D , Vogt M , Sathyanarayanan U , Wagdi A , Riebeling J , Patejdl R and Bruegmann T (2025) Direct stimulation of gastric smooth muscle cells via Gq proteins with light. Neurogastroenterol Motil 37, e70028. 40159839 10.1111/nmo.70028 PMC12075906

[14] Yamashita T , Ono K , Ohuchi H , Yumoto A , Gotoh H , Tomonari S , Sakai K , Fujita H , Imamoto Y , Noji S *et al*. (2014) Evolution of mammalian Opn5 as a specialized UV‐absorbing pigment by a single amino acid mutation. J Biol Chem 289, 3991–4000. 24403072 10.1074/jbc.M113.514075 PMC3924266

[15] McDowell RJ , Didikoglu A , Woelders T , Gatt MJ , Moffatt F , Notash S , Hut RA , Brown TM and Lucas RJ (2024) Beyond lux: methods for species and photoreceptor‐specific quantification of ambient light for mammals. BMC Biol 22, 257. 39538277 10.1186/s12915-024-02038-1 PMC11562817

[16] Govardovskii VI , Fyhrquist N , Reuter T , Kuzmin DG and Donner K (2000) In search of the visual pigment template. Vis Neurosci 17, 509–528. 11016572 10.1017/s0952523800174036

[17] R Core Team (2025) R: a language and environment for statistical computing. R Foundation for Statistical Computing, Vienna, Austria.

[18] Katoh K and Standley DM (2013) MAFFT multiple sequence alignment software version 7: improvements in performance and usability. Mol Biol Evol 30, 772–780. 23329690 10.1093/molbev/mst010 PMC3603318

[19] Capella‐Gutiérrez S , Silla‐Martínez JM and Gabaldón T (2009) trimAl: a tool for automated alignment trimming in large‐scale phylogenetic analyses. Bioinformatics 25, 1972–1973. 19505945 10.1093/bioinformatics/btp348 PMC2712344

[20] Kozlov AM , Darriba D , Flouri T , Morel B and Stamatakis A (2019) RAxML‐NG: a fast, scalable and user‐friendly tool for maximum likelihood phylogenetic inference. Bioinformatics 35, 4453–4455. 31070718 10.1093/bioinformatics/btz305 PMC6821337

[21] Darriba D , Posada D , Kozlov AM , Stamatakis A , Morel B and Flouri T (2020) ModelTest‐NG: a new and scalable tool for the selection of DNA and protein evolutionary models. Mol Biol Evol 37, 291–294. 31432070 10.1093/molbev/msz189 PMC6984357

[22] Abramson J , Adler J , Dunger J , Evans R , Green T , Pritzel A , Ronneberger O , Willmore L , Ballard AJ , Bambrick J *et al*. (2024) Accurate structure prediction of biomolecular interactions with AlphaFold 3. Nature 630, 493–500. 38718835 10.1038/s41586-024-07487-w PMC11168924

[23] Salcedo E , Zheng L , Phistry M , Bagg EE and Britt SG (2003) Molecular basis for ultraviolet vision in invertebrates. J Neurosci 23, 10873–10878. 14645481 10.1523/JNEUROSCI.23-34-10873.2003 PMC2819302

[24] Sakai K , Tsutsui K , Yamashita T , Iwabe N , Takahashi K , Wada A and Shichida Y (2017) Drosophila melanogaster rhodopsin Rh7 is a UV‐to‐visible light sensor with an extraordinarily broad absorption spectrum. Sci Rep 7, 7349. 28779161 10.1038/s41598-017-07461-9 PMC5544684

[25] Tsukamoto H , Chen IS , Kubo Y and Furutani Y (2017) A ciliary opsin in the brain of a marine annelid zooplankton is ultraviolet‐sensitive, and the sensitivity is tuned by a single amino acid residue. J Biol Chem 292, 12971–12980. 28623234 10.1074/jbc.M117.793539 PMC5546036

[26] Yamashita T , Asamoto K , Fujii K , Fujiyabu C , Ohuchi H and Shichida Y (2025) A key spectral tuning site of UV‐sensitive vertebrate non‐visual opsin Opn5. *bioRxiv* . doi: 10.1101/2025.05.19.65487 PMC12405127 40892281

[27] Koyanagi M , Wada S , Kawano‐Yamashita E , Hara Y , Kuraku S , Kosaka S , Kawakami K , Tamotsu S , Tsukamoto H , Shichida Y *et al*. (2015) Diversification of non‐visual photopigment parapinopsin in spectral sensitivity for diverse pineal functions. BMC Biol 13, 73. 26370232 10.1186/s12915-015-0174-9 PMC4570685

[28] Devine EL , Theobald DL and Oprian DD (2016) Relocating the active‐site lysine in rhodopsin: 2. Evolutionary intermediates. Biochemistry 55, 4864–4870. 27486845 10.1021/acs.biochem.6b00478 PMC5305276

[29] Sato K , Yamashita T , Haruki Y , Ohuchi H , Kinoshita M and Shichida Y (2016) Two UV‐sensitive photoreceptor proteins, Opn5m and Opn5m2 in ray‐finned fish with distinct molecular properties and broad distribution in the retina and brain. PLoS One 11, e0155339. 27167972 10.1371/journal.pone.0155339 PMC4864311

[30] Ohuchi H , Yamashita T , Tomonari S , Fujita‐Yanagibayashi S , Sakai K , Noji S and Shichida Y (2012) A non‐mammalian type opsin 5 functions dually in the photoreceptive and non‐photoreceptive organs of birds. PLoS One 7, e31534. 22348098 10.1371/journal.pone.0031534 PMC3279408

[31] Gundelach LA , Hüser MA , Beutner D , Ruther P and Bruegmann T (2020) Towards the clinical translation of optogenetic skeletal muscle stimulation. Pflugers Arch 472, 527–545. 32415463 10.1007/s00424-020-02387-0 PMC7239821

[32] Andrabi M , Upton BA , Lang RA and Vemaraju S (2023) An expanding role for nonvisual opsins in extraocular light sensing physiology. Annu Rev Vis Sci 9, 245–267. 37196422 10.1146/annurev-vision-100820-094018

[33] Leemann S , Kleinlogel S and Schneider‐Warme F (2022) Optogenetic interrogation of cell signalling: human neuropsin (hOPN5) represents a potent tool for controlling the Gq pathway with light. Pflugers Arch 474, 1217–1219. 36319864 10.1007/s00424-022-02765-w PMC9663387

[34] Sun H , Macke JP and Nathans J (1997) Mechanisms of spectral tuning in the mouse green cone pigment. Proc Natl Acad Sci U S A 94, 8860–8865. 9238068 10.1073/pnas.94.16.8860 PMC23167

[35] Yokoyama S , Yang H and Starmer WT (2008) Molecular basis of spectral tuning in the red‐ and green‐sensitive (M/LWS) pigments in vertebrates. Genetics 179, 2037–2043. 18660543 10.1534/genetics.108.090449 PMC2516078

[36] Hagen JFD , Roberts NS and Johnston RJ Jr (2023) The evolutionary history and spectral tuning of vertebrate visual opsins. Dev Biol 493, 40–66. 36370769 10.1016/j.ydbio.2022.10.014 PMC9729497

