# Supplementary material for: Development of visible light‐sensitive human neuropsin (OPN5) via single amino acid substitution
Source: FEBS Lett. 2025 Aug 13;599(18):2612–9. doi: 10.1002/1873-3468.70130 (PMC12457702; doi:10.1002/1873-3468.70130)
Supplement: Supplementary file 1 — Fig. S1. Maximum likelihood tree of Opn5 group. Fig. S2. Estimation of spectral sensitivities for wild type and Lys91 mutants of hOPN5 by a nonlinear optimisation with bootstrap samplings. [file FEB2-599-2612-s001.docx]

**Supplementary figures**

**Fig. S1. Maximum likelihood tree of Opn5 group.** Numbers at the nodes indicate bootstrap support values (≥ 80% are indicated). A scale bar = 0.6 substitutions per site.

**
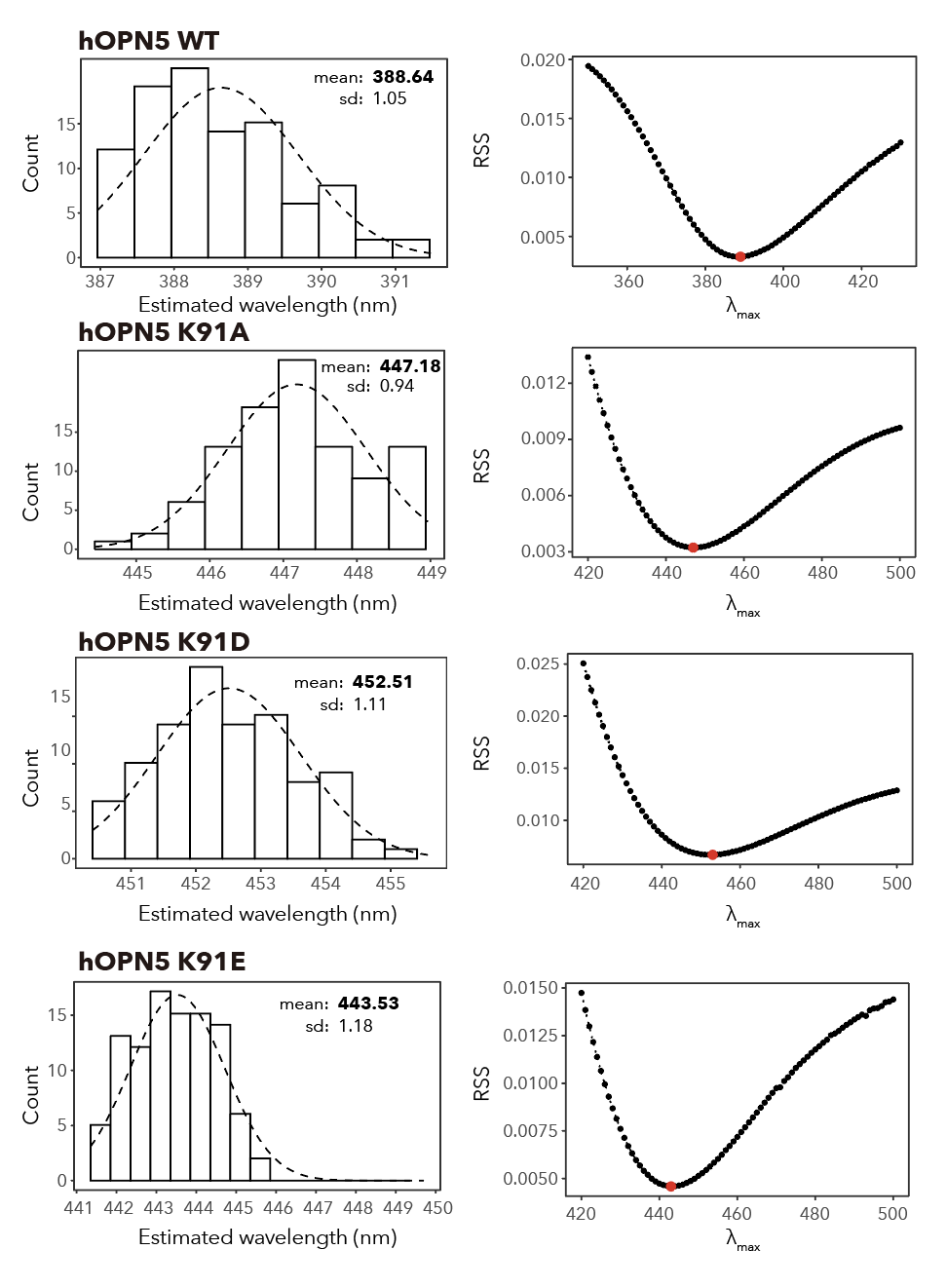
**

**Fig. S2. Estimation of spectral sensitivities for wild type and Lys91 mutants of hOPN5 by a nonlinear optimisation with bootstrap samplings.** (Left panels) Histograms of λ_max_ values calculated from 100 bootstrap samples. (Right panels) Residual sum of squares (RSS) values showing the goodness-of-fit of the 5-parameter logistic curve fitting to the cell responses (response variable) and the effective light intensity (explanatory variable) calculated under λ_max_ values (wavelength ranges: 350 nm – 430 nm for wild type and 420 nm – 500 nm for Lys91 mutants). The RSS value calculated under the predicted λ_max_ for each pigment were shown in red circle.
